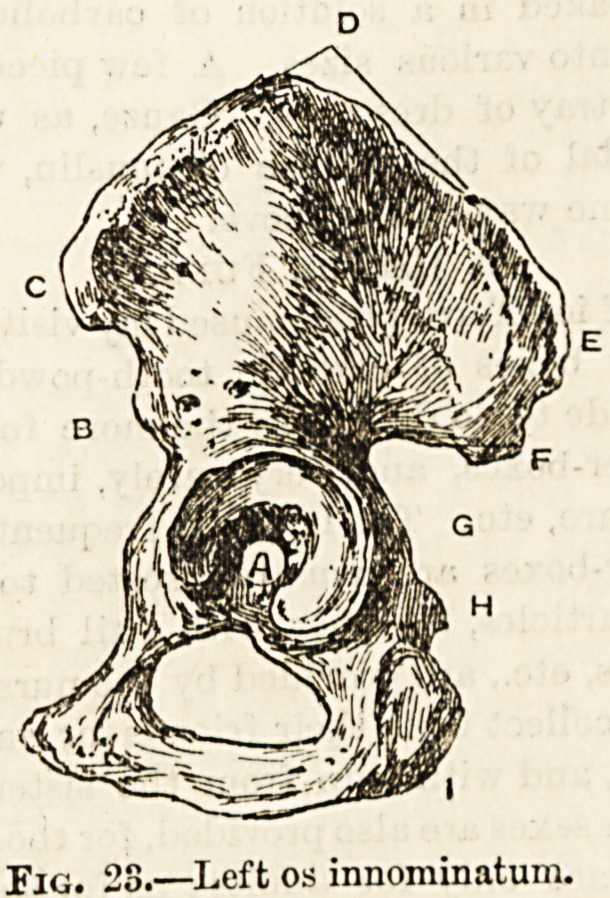# The Hospital. Nursing Section

**Published:** 1902-02-08

**Authors:** 


### The Hospital.

### Nursing Section.

Contributions for this Section of "The Hospital" should be addressed to the Editor, "The Hospital" Nursing Section, 28 & 29 Southampton Street, Strand, London, W.C.

No. 802.—Vol. XXXI. *SATURDAY, FEBRUARY 8, 1902.*

### Notes on News from the Nursing World.

### FOR THE BENEFIT OF ALL TRAINED NURSES.

The necessity of trained nurses sending their appointments for insertion in the Hospital has just been brought home to us in a very striking manner. Within a few days we have received notices of four appointments concerning which no satisfactory evidence as to training has been forthcoming. Reference was made to one of these in our issue of last week. An applicant for a certain post stated that she had been trained at St. Bartholomew's Hospital. This information being officially given us we, of course, published it. Thereupon the matron of St. Bartholomew's, having her doubts, took the trouble to have the books searched for 14 years, and kindly wrote to us that a mistake had been made. Since then the nurse in question has, we are assured, declined the appointment to which she had been elected. In the second case the only "training" of the "nurse" appears to have been at a lunatic asylum; in the third a nurse who had been appointed charge nurse in a sanatorium declined to state where she was trained; and in the fourth, for reasons best known to herself, the nurse begged that her appointment might not be notified. Now it is precisely because we are satisfied that publicity is as the breath of professional life to trained nurses that we take this opportunity of advising them as soon as they receive an appointment to lose no time in sending their names to us for insertion in our list, and also of urging them to inform us if any inaccurate statements are made in the announcements which this list contains. It is clear that persons with insufficient training endeavour, not always unsuccessfully, to obtain positions which should not be given to them; and the only sure way of putting an end to this sort of thing, which does infinite harm to the nursing profession, is for every nurse who has been properly trained to take the fullest advantage of all the publicity that is offered to her. As we intimate elsewhere, no charge is made for the announcement of appointments in our columns, and so long as the name of the school of training is supplied, we are glad to receive and publish them. If fully qualified nurses will only grasp the need of publicity, and personally avail themselves of it, those women who are not fully qualified, and therefore shun publicity, will soon find it impossible to secure posts which they are not competent to occupy.

### IRELAND ASKS FOR A DEPARTMENTAL INQUIRY.

It has been suggested that a committee to inquire into the subject of the nursing of sick poor in Irish workhouse hospitals and infirmaries should be appointed. The proposal was made by a deputation representing the Poor Law Unions and County Infirmaries of Ireland, which recently waited upon the Irish Local Government Board at the Custom House, Dublin, and in particular by Dr. Laffan. The latter urged that such an investigation would alone afford an opportunity for inquiring into and reporting on the best training for the various classes of nurses, the hospitals at which they should be trained, the curricula which should be provided for that training, the examinations to which they should be subjected, and the securities that should be taken that both the training and the examinations should be a reality, and not a sham. Dr. Laffan, in support of the proposal, contended that there is no provision at present to secure any proportion being observed between the number of patients and the number of nurses, and that therefore the proportion has sunk to "a ridiculously low level." However this may be, the departmental investigation asked for in the sister island could do no harm, and might be of great value. It is desirable not to refuse to Ireland concessions which are made to other parts of the United Kingdom, so long as they are possible.

### THE WAR NURSES.

The *Orcana* arrived at Southampton from South Africa last week with the following members of the A.N.S.R. on board:—E. M. Hayden, no leave required, will rejoin the ship; Lady Gifford, home for private reasons, does not return to S. Africa; L. Whiley and D. West, time-expired; Nursing Sister K. Grosvenor A.N.S. was invalided home, but wishes to return to South Africa after leave Nursing Sister Pocock is still reported "dangerously ill."

### "FOR CURRENT EXPENSES."

A very useful as well as handsome contribution has been made by Mr. Howard Morley to the Queen's Commemoration Fund, which, as our readers know, is the organisation for the management and collection of the annual subscriptions to Queen Victoria's Jubilee Institute for Nurses. Mr. Morley has given £1,000 to be employed "for current expenses." It is one thing for people to put down their names as subscribers to a popular movement, and another to specifically help in the prosaic work of paying current expenses. Mr. Morley's timely recognition of the need of money for the latter purpose may induce others to follow his example.

### COUNTY ORGANISATION IN AMERICA.

An interesting organisation of nurses has been formed in Philadelphia, U.S.A. It is called the Philadelphia County Nurses' Association, and members must be graduates of training schools for nurses connected with general hospitals in the county containing not less than 50 beds, and giving not less than two years' training either in hospital, "or where experience gained by post-graduate or other additional nursing work should be considered an equivalent." Graduates from other recognised training schools are eligible, but they must have been resident in the county of Philadelphia for at least a year, and no application for membership is entertained unless it is endorsed by three members who have a personal knowledge of the candidate. Miss Lucy Walker, superintendent of Pennsylvania Hospital, is the president.

### SUBSCRIPTIONS TO NURSING ASSOCIATIONS.

An attempt was made at the last meeting of the Auckland Board of Guardians to put a stop to the annual subscriptions of £10 each to the Bishop Auckland and Crook Nursing Associations. One of the advocates of the motion affirmed that "if the ratepayers subscribed through the Union they were paying twice over for the nursing of the sick poor." This, however, is obviously a fallacy. People cannot be nursed in their own homes and in the workhouse infirmary at one and the same time. Moreover, the persons who are nursed in their own homes are not a burden on the rates. In spite of a vigorous defence of the subscriptions, the motion was only lost by one vote. If it had been carried, the guardians supporting the supposed interests of economy would probably not have been long in finding out that they had made a mistake. As it was urged by an opponent of the motion, the sick poor must be nursed, and if the local nursing associations collapsed through lack of means, the number of patients in the infirmary would be increased to such an extent that the guardians would have to provide extra nurses.

### SOUND POLICY AT ROCHDALE.

The report of the Rochdale District Nursing Association shows a considerable increase in the work done during 1901. In February a nurse was started in the Castleton Moor Ward with two patients. By the end of the year the total she had nursed was 120. The labour during the year had been exceptionally heavy owing to the number of operations at which the nurses had assisted, and in all 16,845 visits had been paid—6,933 more than the previous year. The secretary said it was a matter of great regret that the nurse engaged by the Rochdale Nursing Institute to attend paying patients had been obliged to leave for lack of work. But, she added emphatically, it was quite impossible for the District Nursing Association to undertake cases where the people could afford to pay. There was not only no time for such cases, but the object of the association was, above all, to nurse the sick poor. The association is to be congratulated upon its steady adherence to the policy of tending the sick poor only. That the work is appreciated by those for whom it is meant is shown by the fact that grateful patients contributed over £20, and that over £20 was collected at a fire-brigade demonstration.

### A WARNING TO PRIVATE NURSES.

Under this heading an interesting letter appears in another column, and, as a comment, we would point out that most of the explosions of spirit lamps which occur are due to the wick not fitting. Many of the spirit lamps which are now in common use have large reservoirs, capable of containing a considerable quantity of spirit, and when the spirit gets low capable of containing a considerable volume of explosive spirit vapour, and if between the flame and this reservoir there is the slightest chink not filled up completely by the wick, the flame will be liable to "flash down" into the reservoir, when the vapour will explode with disastrous consequences. If a spirit lamp is well looked after and the wick thoroughly fits the opening provided for it, it is probably a reasonably safe form of heater. But in all the more complicated forms of lamp, especially those in which the spirit is vapourised within a central tube around which a ring of flame is kept burning, if the lamp is not constantly well supplied with spirit the central wick is apt to get charred, and at last to drop down into the reservoir, when there is pretty sure to be an explosion. There is no doubt that one of the safest forms of spirit lamp for warming small quantities of food is the old-fashioned Etna, a conical vessel, to the bottom of which a shallow metal saucer is fastened. Into the latter a measured quantity of spirit is poured and then ignited, and the flame plays around the conical vessel. There being no wick, and the total amount of spirit in use being only about a spoonful at a time, there is but little danger from a "spill," and absolutely none from explosion. The only thing to remember is that if more spirit should be required, it must never be obtained by pouring directly from the bottle, but by placing the required quantity in a spoon and emptying that into the saucer of the Etna. Another very safe form of spirit lamp, and one which will boil a kettle, which an Etna, of course, will not do, is that in which a given quantity of spirit is poured upon a sponge or other absorbent material, generally covered over, and held in place by means of a wire gauze. These lamps are cheap, but they are apt to smell unless carefully managed. Still they are far safer than those with a reservoir.

### A BISHOP ON DISTRICT NURSES.

At the annual meeting of the Chester and District Nursing Association, the report of the Council was adopted. They announced that they had drawn up a new constitution, and a fresh set of rules for the nurses, both of which they hoped would meet with the full approval of the subscribers. The Bishop of Chester, who spoke at the meeting of the added interest which attached to the nursing institution because it was the Daughter of the Deaconess Institution, said that it was impossible to say how great would be the disadvantage to the poor of the city if the nurses were no longer able to be maintained by the Association. The value of nursing had been demonstrated so prominently during the war that it would be paying the worst possible compliment to the citizens of Chester if they were to suppose that the District Nursing Association would be left without support. It had been constantly improving with the object of making the nursing more scientific but not less sympathetic. He thought that it would be a doubtful advance if more strictly scientific methods meant any falling off in the keen personal, sympathetic interest taken in the work by the nurses. They wanted the maximum of scientific skill coupled with the maximum of womanly and Christianlike sympathy with those who were tended. The Association at present employs five nurses for five districts, and during the year they attended 850 cases and paid 23,921 visits. The income for 1901 was £339, while the expenditure was £614. The greater part of the latter was the cost of refurnishing the home and of giving the nurses better salaries, but if the work is to be carried on a further sum of at least £200 per annum will have to be raised to meet the ordinary outgoings.

### THE QUESTION OF TRAVELLING EXPENSES.

In ordinary circumstances the travelling expenses of a nurse who is invited to appear before a Board of Guardians in consequence of her application for a post are, of course, paid. If she is not selected by them, or if she is appointed and accepts the appointment, there can be no objection to the proceeding. But there is some reason for the protest of the Newcastle-on-Tyne Board of Guardians against "young ladies who respond to advertisements in The Hospital" taking a trip to the north at the Guardians' expense and then declining to accept the appointments offered to them. As several of such cases have occurred lately, it is not surprising that the question has arisen. Usually, sufficient details of the duties and conditions can be obtained by letter to enable a nurse to decide whether it is worth while for her to appear before the Guardians; but if she accepts the travelling expenses she might also accept the post if offered to her.

### THE VALUE OF SKILLED SERVICES.

The Belfast Board of Guardians have placed on record their appreciation of the conduct of Nurse Mitchell, the dispensary nurse, who, according to the city coroner was, as the jury and himself believed, the means of saving a human life. The mother of an infant who died at the time of its birth, was in a terrible condition, and her state being brought by one of her children under the notice of a police constable, he obtained the address of the dispensary nurse. It is usual for her to receive premiums from the relieving officer before attending a case, but in this instance, realising the urgency of the matter, Miss Mitchell did not wait to comply with the regulations, but ran with all speed to the home of the woman, whom she promptly relieved. Had she delayed, the coroner said, death would undoubtedly have taken place, and he gave a graphic picture of the condition of affairs. "The surroundings of the poor creature were almost too dreadful for words. She and her infant (the deceased) were lying on the bare boards without any covering, no fire, no light, her other children likewise without bed or covering, all in a state bordering on starvation. The warm-hearted nurse supplied the woman with food and clothing, and subsequently, in conjunction with the constable before referred to, had the whole family removed to the workhouse." The coroner went on to tell the guardians that the dispensary midwives, capable of rendering skilled services, are gradually supplementing the unskilled "handy-women," whose ministrations "too frequently result in disease, misery, and death," and to express the hope that they would appoint nurses of the better type.

### THE NURSES OF ST. LUKE'S HOSTEL.

The Bishop of London, who presided at the annual meeting of St. Luke's Hostel on Monday at the Church House, Westminster, spoke in the warmest terms of the kindness and care of the matron and nurses at 16 Nottingham Place, which he described as an ideal "home of love." He announced that a lady had that day made the generous offer of a hospice for convalescent patients, just outside London, at a cost of £1,600. Subsequent speakers urged the need of a new nursing home, and Canon Utterton said that it was not the patients who suffered, but the nurses, two of whom, to the regret of the matron, were obliged to sleep outside.

### NURSES AND VACCINATION.

The Mile End Guardians have very properly decided to engage twelve additional nurses to cope with the outbreak of small-pox at the workhouse infirmary. We hope that care will be taken to have these nurses re-vaccinated. It is very unsatisfactory to learn that among the victims of the disease are two nurses. This is in striking contrast to the fact that during the epidemic of small-pox at Glasgow, the whole of the nursing staff enjoyed immunity.

### AMATEUR THEATRICALS AT LIMERICK.

An interesting performance of amateur theatricals was given in Limerick in aid of Barrington's Hospital on January 31st and February 1st. The piece produced was Mr. Pinero's comedy "Dandy Dick," and the players were county ladies and gentlemen and officers of the 1st Yorkshire Light Infantry, the Theatre Royal being lent for the occasion by the lessee. A number of the Barrington nurses in uniform sold programmes, and by their untiring efforts must have added largely to the funds.

### INCREASED DEFICIT AT WALSALL.

At the annual meeting of the Walsall Victoria Nursing Institution mention was made of the support afforded to the funds by the various friendly societies in the district, but unfortunately the good example of the Foresters and others was not followed by the well-to-do residents in the neighbourhood, and there was a deficiency of £68 in the income for the past year, to which had to be added £12 on the year before. As the Mayor remarked, "it was not worthy of a town the size of Walsall, with 86,000 inhabitants, only to subscribe £150 per year towards the support of such a noble institution." The reason of the serious increase in the deficit was because the private nursing branch contributed £30 less than during the previous year. The private nursing department, it was stated, had been much hampered by the great scarcity of qualified nurses, largely owing to the continuance of the war in South Africa, though every effort had been made to keep this branch fully equipped. The town clerk did his best to correct an idea, which he said prevailed, that the people who could afford to pay had the first consideration; but the combination of district and private nursing often gives rise to impressions of this kind, and militates against the success of both branches.

### SHORT ITEMS.

A thousand pounds has been contributed anonymously to the Bury branch of the Queen Victoria's Nurses' Fund as "a memorial to the late Queen Victoria." The branch has also received another £500 under the will of the late Miss Harper.—At the annual meeting of the subscribers to the Queen's Commemoration Fund in connection with Queen Victoria's Jubilee Institute for Nurses, on Monday at Londonderry House, the report and balance-sheet for the past year were approved. The fund has handed over to the Institute nearly £3,000.—On Wednesday evening last week Mr. R. Kearton gave his lecture on "Wild Nature's Ways" to the patients and nursing staff of the National Orthopædic Hospital, Great Portland Street, who greatly enjoyed the entertainment.

### Lectures to Nurses on Anatomy.

By W. JOHNSON SMITH, F.R.C.S., Principal Medical Officer, Seamen's Hospital, Greenwich.

### LECTURE X.—THE SHOULDER AND PELVIC GIRDLES.

THE *shoulder-girdle* is the part of the skeleton interposed between the thorax and the upper limb. It is made up by two bones: the *clavicle* or collar-bone and the *scapula* or shoulder-blade (fig. 21).

The *clavicle*, which acts as a stay or prop to the shoulder-blade, and without the support of which the arm would become limp and useless, is a long bone attached at its inner end to the upper part of the sternum and at its outer end to the scapula. This bone presents two curves, which are more marked, as a rule, in the male than in the female. The bone along its inner two-thirds is curved forwards, and along the outer portion backwards. These curvatures, together with exceptional elasticity and resilience of the osseous structure of the clavicle, serve to break the transmitted shock of blows and falls on the shoulder. Still, however, a broken "collar-bone" is often observed in surgical work, particularly in young children, whose bones as a rule are soft and yielding and not liable to be fractured. The frequent occurrence of fracture of the clavicle in the adult may be accounted for by the fact that this bone is composed of very hard and compact osseous structure; and in the cases of young children the break, which is often a partial or as it is called a "green-stick" one, is very probably favoured by the early development of the clavicle, which is one of the first bones in the body to undergo the structural change from soft and yielding cartilage to hard bony tissue.

The *scapula* (fig. 21) or shoulder-blade is a triangular, flat, and expanded bone situated at the back of the thorax and covering here the second and six following ribs. In its relations to the skeleton of the trunk it is almost a completely isolated bone, as the only connection it has with the thorax is by the clavicle. It is embedded in thick layers of muscle, and consequently, except in very thin subjects, only the sides and angles of the bone can be felt beneath the skin. Taking the inner margin of the scapula—that running parallel to the spine—as the base of the triangle of bone, and so making the outer angle the apex, we shall find at this

FIG. 21.—Left scapula: posterior surface.

apex a large pear-shaped depression in the bone. This depression, which is very shallow, is covered by cartilage in a fresh bone and receives the head of the humerus or arm-bone. It is called the *glenoid cavity* (fig. 21A). This shallow cavity, as we shall find out in a subsequent lecture, contrasts very markedly with the deep socket in which is lodged the head of the thigh-bone. The glenoid cavity is surmounted by two large processes of bone, one the *acromion process* (fig. 21B), which is a continuation of a large crest running across the back of the shoulder-blade, which is called the *spine of the scapula* (fig. 21C), and another below and in front of this which is much twisted, and from its resemblance to a crow's beak has been christened the "coracoid" process (fig. 21D). These two processes, together with a connecting bridge of strong ligamentous tissue render an upper displacement or "dislocation" of the upper end of the arm-bone almost impossible, but in consequence of the shallowness of

FIG. 22.—The pelvis.

the glenoid cavity and of laxity of the ligamentous attachments of one bone to the other, the arm-bone is often separated from the shoulder-blade in more or less of a downward direction. If we examine closely the important acromion and coracoid processes and the important shallow depression termed the glenoid cavity we shall be able to form some idea of the meaning of the terms sub-glenoid, sub-coracoid, and sub-acromial as often applied to the different forms of dislocation of the shoulder.

The *pelvic girdle* (fig. 22) is a large and massive girdle of bone placed between the spine and the lower limbs. It (1) serves to transmit the weight of the body to the thigh and leg; (2) to protect certain important organs (womb, urinary bladder, and lower part of intestinal canal); (3) to support the intestines; and (4) to give attachment to numerous large and powerful muscles, some of which enclose the abdominal cavity in front, some keep the body erect, and others move the thigh on the trunk and the leg on the thigh. This girdle, which is a rigid one, is composed of the sacrum and the coccygeal bones behind, and of two large and irregularly-shaped bones at the sides and in front. Each of these bones is commonly called the haunch or hip bone, but it would be well for us to learn to know it by its anatomical name of *os innominatum*.

The *os innominatum* (figs. 22–23), like so many bones in the skeleton, is really a compound bone made up of parts which are separate in the young subject and fused together in the adult. The component parts of this bone, as may be seen in (fig. 22), are three in number: a broad expanded part above and at the side, the ilium (A); the part which assists in closing the girdle in front, the pubes (B); and the thick part behind which supports the weight of the body, the ischium (D). These parts surround a deep socket, called the *cotyloid cavity* or *acetabulum* (C), and the pubes and ischium together enclose a large oval gap known as the *thyroid* or *obturator* foramen (E).

The *acetabulum* (fig. 23, A) may be regarded as the centre of the os innominatum, as the three constituent bones converge here and share in its formation; the ischium taking the lion's share, a little more than two-fifths, whilst the ilium supplies a little less than two-fifths, and the pubes one-fifth. The bony margin of this deep socket is horse-shoe shaped, there being a wide notch below which is bridged over by a ligament, and, under this bridge, allows the passage of blood vessels and a nerve to the interior of the joint. The cartilage lining this articular or joint cavity also takes the shape of a horse-shoe, as the lower part of the acetabulum is formed merely by a thin layer of rough bone which is protected by fat. This large and deep cavity, which is rendered still deeper by the addition of a thick rim of cartilage,

FIG. 23.—Left os innominatum.

affords a secure recess for the head of the thigh-bone, and so helps to form a strong joint which, however, allows much less freedom of movement than the shallow glenoid cavity in the shoulder joint. This is the reason why dislocation of the head of the thigh-bone is so seldom met with, whilst dislocation occurs more frequently at the shoulder than at any other joint.

If we now take a detached *os innominatum* which, it will be seen, is twisted into a shape very much like that of a screw propeller, and follow along its margins, its numerous projections and recesses, we shall be able to review many important anatomical details.

Starting from the body of the pubes, which is joined to the corresponding portion of bone on the opposite side by an adherent disc of tough cartilage, we observe on the upper surface a crest of bone leading to a marked spine called the *spine of the pubes.* Passing along the margin of the acetabulum, we find another spine separated by a concave margin from a superior one, which is larger and more prominent. These are the *antero-inferior and the antero-superior* spines of the ilium (B C, fig. 23). The latter spine, which can be easily felt under the skin, is of much practical importance, as it serves as a fixed point in measurements of the hip and the lower limb. We now follow along the upper part of the bone a broad and rugged edge taking a somewhat sinuous course. This is the *crest of the ilium* (D), to which are attached the large and expanded muscles forming the front wall of the abdomen. Beyond and below this we come upon two more spines which, in contradistinction to the last we noted, are termed the *postero-superior and the postero-inferior spines of the ilium* (E F). If we turn the bone over at this stage of our survey we shall find behind these spines a large surface of rough bone, which is attached by cartilage to the sacrum, thus forming the sacro-iliac joint. Below the lower of the two posterior spines we meet with a very deep notch. This, which gives passage to several muscles, blood vessels, and the large sciatic nerve, is called the *greater sciatic notch* (G). Below this and separated from it by the spine of the ischium is a second and much smaller notch, called the lesser sciatic notch (H). Below this and behind the foramen ovale the bone is much thickened to form the *tuberosity of the ischium* (I), by which the body is supported when we are sitting. We finally pass along a relatively sharp and a sloping margin of bone forming the lower and inner portions of the frame of the foramen ovale and then reach the point from which we started. We must not, however, omit to note that the seat of the union of the two pubic bones in front is termed the *symphysis pubis.*

### Nursing in an Indian Hospital.

By A SISTER.

WE have had several cases of the very distressing form of spinal cord disease known as locomotor ataxy during my work in an Indian hospital, both among Europeans and Natives; they were all men, and all cases of a very exaggerated form. Two cases did very well indeed, both natives, recovering, at least temporarily, almost entire command over their limbs. One of these attended as an out-patient; at first it was necessary to lift him from his carriage on to the stretcher, and carry him upstairs to the suspension machine, to which, after a considerable deal of trouble, he was hung for the prescribed few seconds. When last I saw him, he walked up and down the three flights of steps, leading from the entrance hall to the upper verandah, where the suspension machine was erected, quite easily, without any assistance whatever. He was 45 years old, and very emaciated. Nerve tonics were given, the "suspension" twice a week continued, massage persevered with daily, and a nourishing diet ordered. All the other cases were in-patients, and it was quite a business, on Tuesdays and Thursdays, "the hanging mornings," as we called them, getting the patients upstairs, for we had no lift. The other case, also a recovery, was a young man of 25 years of age; he said that his sister had died of the disease, having wasted away to a skeleton. He himself was little better than one on admission. His joy knew no bounds when one day he successfully carried a brass (wati) basin, half full of water, across the ward without spilling a drop. In both these cases the sight was very little impaired; they were under treatment at the hospital for about five months.

### A VERY SAD CASE.

We had one very sad case, a European; at first the disease seemed to yield to the treatment much more readily in his case than in any of the others; then, as frequently happens, it suddenly made rapid progress, and the patient became all but helpless. After remaining in hospital for four months he was discharged, at his own request, a hopeless invalid. In all hospitals there are sometimes noticeable "runs" of certain cases. The period of which I am writing was certainly our "run" of locomotor ataxy—neither before nor since have we had more than one at a time; then we had six at once, which, considering our small hospital and the comparative rarity of the disease, was a very large number indeed. The same suspension machine is put into requisition when there are plaster of Paris jackets to apply, etc., for spinal curvature.

### CASES OF GUINEA-WORM.

Guinea-worm is of such frequent occurrence among natives that it is difficult to keep up one's interest in the subjects of this painful, though common complaint. One villager was the happy possessor of nine, all of which showed themselves at the same time; he was a mass of dressings. In some places the worm died, and pus forming, the abscess had to be lanced. The discharge is peculiarly offensive. The length of worm in some cases is almost incredible. This man was rather troublesome; during the night he would often remove his dressings, and try to draw the worm out, usually ending by breaking it. Sometimes poultices are ordered to be applied to the swollen part before the worm makes its appearance; but more frequently the "bath" is ordered, and a pad of cotton wool and bandage applied afterwards. When nurses first come out from home they are very much astonished at these cases, and examine them with much interest, hardly believing that they can be of common occurrence; but when they have worked on for a month or two, guinea-worms are regarded rather as a nuisance, filling up a bed which might otherwise contain a very serious case. Our surgical wards present a most peculiar appearance sometimes. If a number of cases of injury to upper or lower limbs, and guinea-worms are in, this is especially so. "Baths" are ordered for nearly every one of these cases, which means that after the dressings have been removed the affected part is to be allowed to soak in hot water and lotion for various lengths of time. Kerosene oil tins are mostly used for leg and arm baths; for the latter purpose raised on a stool. We have a few baths of peculiar shapes which were ordered for very special cases, but for ordinary use we are glad to press any available article into our service, since our hospital is not rich.

### Dressing Here and at Home.

I have spoken of "dressings;" the difference is vast between those used in our small hospital, with its limited means, and the magnificent height to which they have reached at home. Two new arrivals from home were going round my wards with me, and asked about the dressings. When I showed them the trays, which I had prepared ready for the next day, they were both horrified and amused. There was one case, a gunpowder accident; the hand was very much injured, and it was found necessary to amputate the fingers as far as the last digit. I went on to explain that all dressings were removed under an irrigation of carbolic lotion (1 in 20) and hot water; after the wound was thoroughly cleansed, if there were no sutures to remove, the part was gently dried with "antiseptic chindi" (which I will explain later), and in this case iodoform was then dusted on, pieces of lint spread with vaseline were wrapped round each digit, a little tow, a splint and bandage applied, and the limb replaced on its cushion. There was the chart hanging up over the bed, and a better one could not have been desired under the circumstances; the highest record of temperature was 102°; the accident had happened some miles away, and the patient had been suffering from considerable shock on admittance. My companions were obliged to admit that our extremely simple method of dressing was very successful indeed. We then visited all the amputations; some were dressed with iodoform, some with boracic, either in the form of powder or ointment, if with powder a piece of lint wrung out in lotion, then cotton or tow, was all that was applied. All were quite satisfactory, and had very good charts. Both these nurses have now worked with us for over a year, and have long since fallen into our ways, though they often yearn for the handy little iodoform pots, the unlimited supply of dressings, etc., to which they have been accustomed. A certain allowance of lint, gauze, wool, tow, bandages, etc., iodoform and boracic powder is indented for every day by the charge nurse; this is countersigned by the house surgeon, but when there are several dressings to be renewed three and four times a day it is quite impossible to make the allowance suffice, and then there is nothing for it but to go to the office and interview the house surgeon.

### The Horrified London Nurses.

One of the new arrivals had been a district nurse, and therefore did not find the new life so difficult as the other one, who came from one of the largest hospitals in London, where the supply of everything is on a lavish scale. A thing which horrified them both was that poultices were made not on tow but on cloth, which after being removed, was washed thoroughly, dried, and put away for future use. Of course this is not allowed when there is any discharge of pus, but is always carried out in cases of pneumonia, or other unbroken surfaces, for which the poultices may be ordered. "Antiseptic chindi" is simply any ordinary soft material, old damask, table linen, etc., sent to the hospital. This is boiled, then soaked in a solution of carbolic acid lotion, dried, and cut into various sizes. A few pieces of this are placed in every tray of dressings. Gauze, as used by us, is simply a material of the texture of muslin, which is prepared in the same way as the above.

### A Useful Fund.

The variety of iodoform pots amused my visitors. In some wards they saw boxes of carbolic tooth-powder, with perforated lids, made to do duty. In the more fortunate ones, little tin pepper-boxes, and, very rarely, imposing ones of glass, earthenware, etc. The latter so frequently get broken that the pepper-boxes are usually resorted to in the end. Most of these articles, together with nail brushes, enamel bowls for lotions, etc., are provided by the nurses out of any sums they may collect from their friends for ward use. From this same fund, and with help from the sisters, clothes for children of both sexes are also provided, for those supplied by the authorities are only for adults; as for the women, the "saries," with their numerous windings and folds, are quite impossibilities for bedridden cases; and petticoats and jackets are supplied. Among native men and women, Christians excepted, it is considered very bad manners to have the head uncovered, so for these are given printed red cotton handkerchiefs, or old saries are torn into squares and stitched round.

### Newcomers Discouraged.

In mentioning the horror of our new nurses respecting the washing of poultice "chindi," I omitted to add their equal astonishment when told that all bandages (if at all passable) are washed and rewound ready for use. They are got up very well indeed. Once a week all the old bandages are called in and new ones issued. A very strict account of these is kept. It takes some little time to get used to all the arrangements, and the courage of some of our newcomers leaks out, and they return home, thoroughly discouraged and upset. We lost a splendid nurse in this way; she had been sister of a large ward in a London hospital, supplied with all the accessories of hospital life ready to her hand, and she felt that she could not do without them. Several of us pleaded that she would stay with us for the sake of the hospital we all loved so well, for had we not seen it improve yearly? But it was no use, she could not settle, and went back to England and resumed her old position.

### To Nurses.

**We invite contributions from any of our readers, and shall be glad to pay for "Notes on News from the Nursing World," or for articles describing nursing experiences, or dealing with any nursing question from an original point of view. The minimum payment for contributions is 5s., but we welcome interesting contributions of a column, or a page, in length. It may be added that notices of appointments, entertainments, presentations, and deaths are not paid for, but that we are always glad to receive them. All rejected manuscripts are returned in due course, and all payments for manuscripts used are made as early as possible after the beginning of each quarter.**

### Fever Nursing in its Relations to Fever Hospital Administration.

By J. T. C. NASH, M.D.Edin., D.P.H.Camb., late Demonstrator in Bacteriology, King's College, London; Medical Officer of Health, and Medical Superintendent Borough Sanatorium, Southend-on-Sea; Resident Physician, Brighton Fever Hospital; Lecturer on First Aid and Home Nursing, London School Board.

THE physician responsible for the administration of a fever hospital may in my opinion be greatly helped if his nursing staff be intelligently acquainted with the principles of isolation and disinfection. The application of these principles may differ in different fever hospitals, but the principles remain the same, and to appreciate them no special knowledge of medical diagnosis is required; indeed, their application as far as a nurse is concerned is quite independent of the diagnosis of disease. It would prove of advantage if every new admission to a fever hospital were looked upon as a doubtful case by the nurse until the patient has been seen by the doctor. In this way I am sure the risk of disaster would be reduced to a minimum.

To effectively carry out isolation and disinfection all that is necessary to be borne in mind is that each infective disease is supposed to be the result of the implantation of a particular kind of germ or bacterium (different in each disease) in the body of a susceptible person. It is not necessary for the nurse to know the name or natural history of the germ, nor to understand the pathological effects produced by its gaining access to the human body. To practise efficient isolation and disinfection, it is only necessary for her to grasp the meaning of the germ theory of disease; to believe that this has been demonstrated to be a fact in certain diseases, and to be logically true for all infective diseases. She must then understand in what way germs are supposed to gain access to the human body and what are the various means taken to prevent their spread from one person to another, and how they may be rendered harmless or destroyed—on such knowledge is founded the theory and practice of isolation and disinfection. Disease-producing germs may exist in the soil, in air, in water, in milk, or other foods, etc. Through one or more of these agents disease may be acquired, and public health measures are directed towards ascertaining if any such possible source of contagion exists and then combating it. But when once a case has occurred, infection by personal agency becomes the commonest source of fresh infection, and may occur either (*a*) directly from the sick to the healthy, or (*b*) indirectly through a third person or through the air or fomites. In former days diseases were classed as either contagious or infectious, according as to whether they were supposed to be acquired directly from actual contact with a patient or indirectly through the air or by means of a third person or by fomites; but no such distinction is recognised nowadays, all zymotic diseases being classed together, and the possibility of transmission, both directly and indirectly, acknowledged in all cases.

Direct transmission is obviously the most likely method of the spread of an infectious disease. An infected patient coming into direct contact with susceptible persons, the germs of disease have but a short way to travel, and it is easy to understand how they may be transplanted by direct contact. Of all the kinds of contact kissing is obviously one of the most dangerous; so too are such practices as are common to children of sharing, say, a sweet in common by sucking it in turns, or an apple by alternate bites, or feeding each other with a common spoon, etc. Almost as dangerous is the use of common toys, or the holding of hands in games, etc., for by these means germs may easily pass from the infected to the healthy. The sharing of a common-room or ward is certainly not free from risk, under ordinary circumstances, but if (*a*) the hygienic work of a ward or room is efficiently carried out, and the children are kept in bed with sufficient air space around each bed, and (*b*) the nurse in charge thoroughly understands that she is the most likely carrier of infection and takes all reasonable precautions to anticipate and prevent this risk, I think this risk may be very considerably diminished. Of course, in infectious diseases accompanied with sneezing, cough, or desquamation the chances of aërial infection apart from personal conveyance are greatly increased, and are, indeed, matters which must ever be kept in mind and appropriately dealt with. Even talking and crying on the part of patients increase the element of risk when the disease germ is present about the mouth or throat.

To illustrate these points,

(1) *Enteric or Typhoid Fever*, which is a definitely infectious disease of undoubted bacteriology, may as a rule be safely treated in a general hospital if precautions are taken to disinfect the stools and urine in the proper way. If there is any concomitant pneumonia the sputum must be similarly treated. After attending to a typhoid patient the nurse is instructed to carefully disinfect and wash her hands before touching another patient or taking a meal herself. The reason for this is that the excretions are infective, and contain the disease-producing germs. As long as the excretions are liquid, the bacilli are imprisoned in the fluid and cannot escape and endanger others but if any splashing occurs, or if cleansing after each evacuation is not thoroughly and conscientiously carried out, particles dry on the bed-clothes or person, are then no longer imprisoned by fluid, and are thus readily transferred to the nurse when she smooths the sheets or attends to the patient otherwise: or the nurse may carry them to another patient, or they may occasionally even be air-borne. If, however, sheets are not allowed to get soiled, or if soiled are immediately changed, not allowed to dry, but placed at once in a disinfectant fluid, and if the nurse is always careful to disinfect her hands immediately after attending to a patient, I think the risk of infection from a case of typhoid, even in a general ward of a hospital, is reduced almost to nil. Under enlightened treatment a secondary case of typhoid in a hospital should be of extreme rarity.

(2) *Diphtheria*.—In this disease the pathogenic organism is strictly localised, being only found, as a rule, in the throat or nose. These parts being usually moist, the microbe is not dislodged during quiet breathing, and an ordinary case of diphtheria might be treated in a general ward without risk, provided the child is kept apart from the others, in a well-separated bed, the nurse exercising extraordinary precautions by wearing an overall when attending to the infected patient (this being removed on leaving his bedside), and carefully disinfecting and washing her hands before attending to other patients. If, however, the child sneezes or coughs or spits about, the risk of infection is greatly increased, for the germ may easily be carried in particles of mucus, etc., which, getting dry, may be carried in the air to other patients. For this reason it is better to treat diphtheria in separate wards, but even where there is sneezing, cough, etc., if a little cubicle were used, such as I shall immediately describe, any case of diphtheria could, in my opinion, be treated in a general ward with ordinary precautions; but this would, of course, be justified only if a separate ward is not available.

(*To be concluded next week.*)

### Sick-Room Cookery.

By Maude Mason, Principal of the Bradford School of Cookery.

### SOLID FOOD.

We will now consider that the patient is strong enough to take more substantial and varied food than "a little fish," and I think the first kind which suggests itself is chicken. This is for the good reason that the fibres are shorter, more easily broken up and digested than beef or mutton. Of the two latter mutton is usually considered more digestible than beef, though it does not contain as large an amount of proteid.

### The Right Way to Boil.

Boiled chicken is usually given in preference to roasted, though the breast of roasted chicken may be given when the digestion is a little stronger. By referring to the rules given for cooking fish, it will be readily admitted that the chicken should be placed in boiling water, it should then be allowed to boil quickly for a few minutes, and then cooked gently till tender, about 1 hour or 1¼ hour. When any kind of food has to be boiled it is a great mistake to imagine that the water must boil furiously—an infinitely better result is obtained if the water be merely allowed to simmer, or as the French have it, "to smile." A little lemon juice squeezed over the breast keeps the chicken whiter, and it should be wrapped up in a cloth to prevent bits settling on it. The white sauce given in "Fish Cookery" is appropriate for serving with it. Young partridge and hen pheasant also yield tender food.

### A Chicken Panada.

Another appetising dish may be made as follows:—Take 4 oz. of breast of raw chicken, place it in a jar with a little salt, no water. Cover tightly and stand in a saucepan of boiling water, let the water boil for two hours, then pound the flesh and pass it through a sieve, adding some of the liquor from the chicken; add one tablespoon of cream and warm carefully, or it may be served cold. If there is only one patient to cook for, one chicken may be made to do duty on several days if the weather be favourable. Serve the breast the first day with white sauce; the second day make some chicken creams—pound some of the cooked chicken, and add half its weight of bread previously soaked in a little milk, squeezed dry and just cooked over the fire fire with a tiny piece of butter, season with salt and pepper, add a little cream, and steam in a buttered mould or cup. A tomato cut up and cooked in a little butter, passed through a sieve to remove the skin, and seeds would be delicious served with it.

### Chicken Rolls.

The remainder of the meat may be made into chicken rolls. Chop it up finely and add it to a very stiff sauce made according to previous directions, but with the following ingredients: 1 oz. butter, 1 oz. flour, ½ pint milk and chicken stock mixed, some seasoning, or a little minced ham or tongue may be added. Turn all on to a plate to get cold and firm, shape it into rolls, dip each one separately into beaten egg and bread crumbs as for fish, and fry them—then drain and serve hot. If the invalid is not allowed fried food, make a similar mixture, but rather thinner, turn into a pie dish, sprinkle some brown crumbs over, and put into the oven to get hot through. If no tongue or ham be used, it would be a great improvement to cook a little onion and mace in the stock, removing them before using it for the sauce.

### Sweetbread and Tripe.

Sweetbread (the thymus and pancreatic gland of the calf) is rather an expensive dainty in towns, but it is very digestible and suitable for an invalid. To cook it, first soak in cold water, then just boil, and put again in cold water to cool; cut away any pipes attached, then cook it very gently in hot white or brown stock, with onion and other vegetables added, till tender (about half an hour). Remove the vegetables and thicken the gravy. Tripe is also easy of digestion if the fat has been extracted before cooking. It is nice cut up into squares, and cooked in milk with onion. When tender the onion should be cut up finely and returned to the milk; thicken the milk with flour, add seasoning, and serve all together. It may also be dipped in batter, or egg and bread crumbs, and fried.

### The Fringe of the Subject.

In these short articles I have only been able to touch the fringe of the subject of "sick-room cookery," but I shall rest satisfied if they have proved of use to some. May I bring them to a conclusion by quoting the words of Epictetus, in which all who have entered the arena of the world's work may find encouragement and stimulus—"As the sun does not wait for prayers and incantations that he may rise, but shines at once and is greeted by all, so neither wait thou for applause and shouts and eulogies that thou mayest do well, but be a spontaneous benefactor, and thou shalt be beloved like the sun."

### Greenwich Guardians and Nurses.

### A STORM IN A TEA-CUP.

At the meeting of the Greenwich Board of Guardians last week, the clerk read a letter from the medical officer, in which he begged to contradict the report that a fancy dress ball had been held at the Infirmary. A letter of protest had also been received from the matron on behalf of the nurses to the following effect:—

January 22nd, 1902.

To the Chairman, Ladies, and Gentlemen of the Infirmary Committee.

The matron, on behalf of the nursing staff, wishes to call the attention of the Guardians to the injury done to the good name and prospects of the nurses by the question asked at the last board meeting on account of the publicity given to it by the press.

Therefore the matron wishes to ask the board whether in future questions concerning the conduct and character of any or all of the staff may be considered in committee, so as to avoid publicity being given to accusation of fault before such have been proved.

It had been placed upon the minutes of the infirmary committee, that while regretting the episode, the committee were very pleased that the nurses should have had some light and harmless amusement.

The Chairman of the Infirmary Committee, Dr. W. Fox Batley, said he was sure the board would be pleased with the mild and proper form in which the matron had worded her protest. The statements that had been made in the public press had given a vast amount of pain to the nurses, who were all ladies of very respectable connections, some being the daughters of clergymen. They were most indignant that such a report should have been spread over the whole of the United Kingdom, and he sincerely hoped that the members of the press present would deny—(at this point there were interruptions)—that any such ball took place. He hoped that, as their gentle, kindly matron had suggested, any member wishing to make inquiries or complaints, or to point out anything wrong in connection with the nursing staff, would be kind enough to do so to the committee, so as to avoid such statements as had been made being rushed into print without respect of truth.

A Guardian said he very much deplored the fact that the statement alluded to should have become of almost national interest. He believed the question was originally asked in a frolicsome mood, and not seriously; he was very sure that none of the Guardians looked upon it seriously.

Another GUARDIAN said they could not help the publicity, since the representatives of the press were allowed to attend the meetings of the board. Nobody wished to suggest that the nurses intended to do anything wrong.

After remarks from one or two guardians, of which the rest of the Board plainly showed their disapproval, a speaker suggested that the nurses might be allowed to play Ping Pong as a harmless amusement.

The Rev. Father SHEEN, who was applauded, said he should have thought no more need be said about it, except to remove any unpleasant feeling. The feelings of the infirmary committee had been deeply ruffled; the question was considered to have affected the right conduct of the establishment. The matter should now be allowed to drop. Any further statements would do harm, and would simply have the effect of probing the wound.

Mr. VERCOE ABBOT, who originally put the question at a former meeting, then made a speech in which he said he had hoped that after what had happened in the press, the matter might have dropped. He had intended laying the case before the Local Government Board; this, however, he would not now do. He still held that a guardian had a right to ask any question of the Board. The question was innocently asked. He would be the last to get up and endeavour to curtail the recreations of the nurses, but he did and would protest against the vehement speeches that such a question evoked. There had not been a single denial given that some of the nurses were dressed in male costume, and it was most improper.

One of the Lady Guardians said "It was in their private room," to which Mr. ABBOT replied by quoting from the orders of the Local Government Board in respect to dancing in rooms belonging to the union buildings. He said he could not bear the idea of old people dying in the ward, their last moments, before passing into another sphere, disturbed by the noise of the nurses downstairs. Sounds could be plainly heard in the ward above.

"Kilts?" a Guardian suggested.

"Oh! I say nothing about that," Mr. ABBOT replied; and added that when people talked about taking away the good name of the nurses, it was the kind of claptrap which he would expect to hear in a public-house bar.

The Rev. H. S. HILLS gave a very emphatic denial to the statement than any fancy dress ball whatever took place in the infirmary. The last speaker had said he had intended taking the whole matter before the Local Government Board, but he had decided not to do so.

Mr. ABBOT, interrupting, said he had been asked not to, at which some of the Guardians laughed.

"I beg leave," Mr. HILLS continued loudly, "to give a most emphatic denial to the statement,—There was *no* fancy dress ball, there was not a single man in the place."

Mr. ABBOT: "There were some dressed as men."

Mr. HILL: "It is a perfect insult to ask what our nurses do in their recreation time; you will be asking what they wear, next. I ask to have it published far and wide that there was no fancy dress ball."

Even then Mr. Abbot was not satisfied. He wanted to know how Mr. Hills knew that there was not a single man present.

A GUARDIAN said: "We have it from the matron"; and Mr. HILLS added: "Miss Dixon told me so."

After the discussion had lasted half an hour the CHAIRMAN interposed. The nurses, he said, were in their own room. Miss Dixon was present. She was one of the most severe disciplinarians he knew, and a very good woman He should have thought that the very fact that the nurses had her sanction and approval in their amusements was enough to show that the report that had been spread was an exaggeration. It had been the source of great discomfort, and he thought the results had shown that such questions should be asked in camera. The statement that had been made conveyed an innuendo that the nurses in that establishment were behaving in an unseemly way. It would be impossible to retain ladies on the staff if more care were not taken of their reputations.

Again Mr. Abbot tried to speak; but the CHAIRMAN, remarking that the resources of civilisation were not yet exhausted, put the minutes of the Infirmary Committee, and they were carried by an overwhelming majority.

### Appointments.

[No charge is made for announcements under this head, and we are always glad to receive, and publish, appointments. But it is essential that in all cases the school of training should be given.]

ALTON UNION INFIRMARY.—Miss Evaline M. Stansfield has been appointed superintendent nurse. She was trained at St. George's-in-the-East Parish Infirmary for three years, where she was afterwards charge nurse. She has since done private nursing.

CHELSEA HOSPITAL FOR WOMEN. — Miss Katherine Lundie has been appointed night superintendent and assistant matron. She was trained at St. Bartholomew's Hospital, London, where she has been four years. On several occasions she has had charge of the wards in the absence of the ward sisters during holidays.

CITY FEVER HOSPITAL, SHEFFIELD. — Miss Charlotte Richardson has been appointed charge nurse. She was trained at the Royal Lancaster Infirmary.

CITY HOSPITAL, PARKHILL, LIVERPOOL.—Miss M. A. Pearson has been appointed assistant matron. She was trained at Manchester Royal Infirmary. She has since been night superintendent at Nottingham General Hospital, and for the last five years has been sister at Manchester Royal Infirmary. Miss Pearson has also had some fever experience at Leeds Fever Hospital.

DUNDEE PAROCHIAL HOSPITAL.—Miss Margaret C. Fraser has been appointed charge nurse. She was trained at the General Hospital, Leith, for three years, and has since been district nurse at Perth.

GRAVESEND AND MILTON UNION INFIRMARY. — Miss Minnie Etherington has been appointed nurse. She was trained at St. Pancras Infirmary, where she has since been assistant nurse and charge nurse.

KING'S NORTON UNION INFIRMARY.—Miss Mary Elizabeth Cave has been appointed sister. She was trained at Birmingham Infirmary, where she was afterwards charge nurse. She has since been assistant nurse at Plymouth Union Infirmary. Miss Cave holds the L O.S. certificate.

LONDON SCHOOL BOARD.—Miss M. Florence, St. George's Nursing Home, Streatham Hill, has been re-appointed lecturer on home nursing for Wandsworth, and has been appointed lecturer for Streatham for the present course. She was trained at Leicester Infirmary, and subsequently belonged to the Leeds Trained Nurses' Institution for several years. She has also nursed in Brussels and Paris.

MACCLESFIELD GENERAL INFIRMARY.—Miss Nellie Berry has been appointed night sister. She was trained at the Preston and County of Lancaster Infirmary, and has since been sister of children's wards at Macclesfield Infirmary.

MERCER'S HOSPITAL, DUBLIN.—Miss C. Mackay Connon has been appointed night sister. She was trained for three years at the Royal Aberdeen General Infirmary, afterwards holding the post of sister of the Ophthalmic department and holiday sister of a medical flat. She has also been charge nurse at the Mirfield Memorial Hospital, night superintendent at Manchester Children's Hospital, Pendlebury, and for some years Queen's Nurse in London and the provinces. Miss Connon holds the L.O.S. certificate.

Mexborough Cottage Hospital.—Miss Annie Roote has been appointed matron. She was trained at the London Hospital and at the Melbourne Hospital, and she was for some years nurse at the London Hospital; staff nurse, surgical and accident ward sister in charge of the theatre at Westminster Hospital; and matron of the Sanatorium Royal Masonic Institution for Boys, London, N.

National Hospital, Queen's Square, Bloomsbury.—Miss M. Girdwood has been appointed night sister. Miss Girdwood was trained at St. Mary's Hospital, Paddington; she afterwards held the post of out-patient sister at the Children's Hospital, Queen's Street, Belfast, and later on that of night sister at the Children's Hospital, St. Michael's Hill, Bristol.

Paddington Green Hospital for Children.—Miss Katherine Reeve has been appointed home sister. She was trained at St. Bartholomew's Hospital, London, and the East London Hospital for Children. She has since been night superintendent and assistant matron at Chelsea Hospital for Women.

Scarborough Union Infirmary. — Miss Mary Ann McArdle has been appointed superintendent nurse. She was trained at Staffordshire Institution for Nurses, Stoke-upon-Trent, and was afterwards staff nurse. Previously she was for seven years charge nurse of the epileptic and refractory wards at the County Asylum, Park Side, Macclesfield.

Swansea Hospital.—Miss Florence Steggall has been appointed sister. She was trained at Kensington Infirmary for three years, and was afterwards for a year in charge of the children's ward. She has since for 18 months been sister of the children's ward at Burton-on-Trent Infirmary.

Throne Convalescent, Consumptive and Children's Hospital, Belfast.—Miss Emily J. Mildred has been appointed matron. She was trained at Leeds General Infirmary, where she was afterwards sister for three years. She has since been matron of Grantham Hospital.

Wellington and District Cottage Hospital, Somerset.—Miss Mabel Brooks has been appointed matron. She was trained for one year at the Wellington Cottage Hospital, and for three years at Crumpsall Infirmary, Manchester, where she was also ward sister for two years. From January, 1900, up to the present time, she has been matron of the infirmary at The Leys School, Cambridge.

Woodlands Convalescent Home, Mawdon, Leeds.—Miss Maud M. Walker has been appointed matron. She was trained, for three years, at Dundee Royal Infirmary, and has since been on the staff at St. John's House, Norfolk Street, Strand, at All Saints Boys' Orphanage, Lewisham, and she has also been working with the All Saints sisters at different homes. She has been locum tenens superintendent district nurse at Morley, Leeds. Miss Walker holds the L.O.S. certificate.

### Where to Go.

London School Nurses' Society.—A dramatic performance in aid of the above entitled "Candida; a Mystery" by G. Bernard Shaw, will be given on Saturday, February 15th, at the Cripplegate Institute, Golden Lane, E.C., at 3 p.m. The incidental music will be performed by members of the Stock Exchange Orchestra. Reserved seats 1s., 2s., and 5s each may be obtained from Miss Honnor Morten, Ivy Hall, Richmond.

### Queen Victoria's Jubilee Institute.

### MANSION HOUSE MEETING.

On Thursday last a meeting was held in the Long Parlour at the Mansion House "to consider what steps should be taken to make the collection from the City towards the Women's Memorial to Queen Victoria a thorough success." The Lady Mayoress took the chair, which was placed at the end of a long table, gay with two large épergnes filled with white lilac and orchids. She was supported by the Marchioness of Londonderry and the Duchess of Somerset, while grouped around were Viscountess Knutsford, Lady Mary Howard, Lady Edmund Talbot, Lady Samuel, and others.

The proceedings were a little late in beginning, owing to the unavoidable detention of Lady Londonderry, and it was rather amusing to hear the efforts of one lady amongst the audience, who had a good grasp of the subject in hand, trying in a whisper to explain to some friends near her what they had come to the Mansion House to do. Both these ladies were most anxious to put their shoulder to the wheel and work well, but neither seemed at all certain as to the purpose for which the money they were going to ask for was wanted, nor the *raison d'être* even of the Queen Victoria Jubilee Institute for Nurses. By the time the meeting was opened, thanks to the explanation of the better informed acquaintance, and to the sheaf of literature placed on each chair, these ardent workers had a better understanding of the subject.

The Lady Mayoress—who has a clear voice and speaks well—explained that at the meeting which she had held at the Deanery in July last it was suggested that for the purposes of collecting throughout the City it should be divided into wards, the wife of each alderman undertaking to organise the collection in the particular ward with which she was associated. In many cases this suggestion had been responded to, but in others the ladies had found it impossible to do as they had been asked. Therefore the present meeting had been called so that matters might be put upon so firm a basis that the collectors could show their loyalty to their late Queen by hard work and by realising a considerable sum.

Mr. Harold Boulton, the honorary secretary, then gave a sketch of the rise and the growth of the Queen's Institute, and related how he had pleaded the good cause in which they were now interested in town halls, municipal buildings, assembly rooms, etc., all over the kingdom till, at last, he was proud to find himself speaking in the "Mecca of all charitable pilgrimages," the Mansion House of the City of London. Two appeals, he said, had already been made for this fund, one addressed to men and issued by the Archbishop of Canterbury, Cardinal Vaughan, the Duke of Portland, etc.; the other to women, sent out by the Marchioness of Londonderry and other ladies, including Miss Florence Nightingale. He was pleased to be able to report that already £20,000 had been received, and that roughly speaking about £5,000 more had been promised. He thought that he might say with certainty that £40,000 to £50,000 would ultimately be raised, but no doubt his hearers would agree with him in hoping that this memorial might reach the same sum as that originally given by the women of Great Britain to Queen Victoria, viz., £70,000. This, he maintained, was not by any means an impossible scheme, for, though the one square mile of which the City of London was composed was a difficult area to work, owing to the fact that the day population was so large and the night population so small, he was sure it would respond nobly to the call. Letters were often received at the office saying how comparatively easy ladies found it to collect for this fund. The rich gave of their abundance, but the poor were equally delighted to contribute their pence. This was not only because there was a genuine and abiding love for Queen Victoria in their hearts, but because they also felt the money given to the fund might ultimately come back to benefit them.

The LADY MAYORESS, at this stage, read a telegram from the wife of the rector of All Hallows, London Wall, stating that the scheme had been laid before the 300 factory and work girls sheltered each morning in that church, and it had received their cordial support.

Lady LONDONDERRY announced that nine-tenths of the counties were now thoroughly organised, and the partial organisation of the others was in progress. The collection was not yet half finished, but at present the best county was Lincolnshire, which had sent £1,500, and the best borough, Paddington, with £1,400, whilst Hertfordshire had contributed £1,200, and Surrey, too, would probably stand well. Some of the colonies were beginning to send contributions, but too much should not be expected from them, as the larger ones had their own nursing systems. A letter received that morning from Government House, Accra, showed the deep and practical sympathy with the scheme evidenced by the native women and girls of the Gold Coast. When first it was proposed to collect from these women, the authorities thought that it would almost be futile, as many were so poor, but the result had been wonderful, £25 8s. 9d. having been forwarded from the Gã women and girls, and £8 10s. from the Hausa women and girls. Lady Londonderry added that she had that afternoon heard of £33 10s. for the memorial from the young women employed in the Savings Bank Department of the General Post Office.

After a short speech from the Duchess of SOMERSET, and a vote of thanks to the Lady Mayoress, the ladies present, who represented the London wards, met privately to discuss the most effective way of carrying out their various schemes.

### Nursing on the "Princess Christian Hospital Train" at Pretoria.

BY AN ARMY RESERVE SISTER.

IT is now two months since I received the appointment for duty on the Princess Christian train. As the readers of THE HOSPITAL know, it was sent out to South Africa by the "Central Red Cross Committee," Princess Christian supplementing the £6,100 raised by the borough of Windsor toward the cost of the train by a donation of £650, the balance of a Red Cross fund which was invested in Her Royal Highness's name after the Soudan campaign of 1885. The object of the train is not merely to provide means of conveying the sick and wounded from the front, but also to have a perfect self-contained hospital on wheels. The train is painted white both inside and out; on either side of every carriage the centre panel has a conspicuous red cross painted on white ground with the words "Princess Christian Hospital Train" in royal blue and gold. It has a very bright appearance, for the orderlies keep it beautifully clean, and, like sailors when they arrive in port (only this is usually at a siding in a station), they scrub and wash so as to keep it smart and in good order. Major Morgan, who has charge of the train, takes great pride in having it spotlessly clean, and has the reputation of having the best-kept train in the service. The Red Cross Society has now turned it over to the Government. Our senior sister has been with the train from the commencement of its career, now nearly two years. We make frequent trips to Durban with convalescents who take ship there for home. Our last wounded from the field were from Colonel Benson's column, when 104 men and 8 officers were brought up from Springs.

### ILLUSTRIOUS VISITORS.

The train from time to time has been honoured by many illustrious visitors, Lord and Lady Roberts, with daughters, Lord Kitchener, and General Baden-Powell. Also our late lamented Prince Christian Victor visited it several times, and was most interested in the work. Again, when at Durban, the Duke and Duchess of York passed through it, inspecting every detail and expressing their pleasure at having the opportunity of visiting it. The Princess Christian train commenced duty on March 29, 1900, and was the first to pass over the trestle bridge at Colenso, also to enter Ladysmith. Many a Tommy will never forget its welcome appearance when it arrived to take them away from that scene of suffering and death. A special Providence has seemed to watch over it, for no serious accident has ever occurred. Once it had a narrow escape, the bridge at Ingogo being blown up 20 minutes after it had passed over; and although the train itself was not attacked, yet the staff was one night in danger of the Boer bullets fired at the garrison at "Val." Without losing any time the gallant major conducted the sisters to a place of safety within the fort. A party of Boers were endeavouring to cross the line, and our troops had an encounter with them in trying to prevent it, but in the darkness of the night they succeeded in getting across, and all was quiet again. Although we knew the Boers would never blow up a hospital train, yet our train runs the same risk as others, for the mine may be laid for a provision train when a hospital train comes along unexpectedly.

### THE BOER PATIENTS.

We frequently carry wounded and sick Boers, and they have the same care and attention as our own invalids. The British soldiers are always especially interested in them, and try to make them talk, and I have never seen an unkind action done to them. Nor do the captured Boers evince any dislike to their captors; when once they give up, the bitterness seems to vanish and they are friends. Since the commencement of its career the Princess Christian train has carried 8,948 patients and travelled 51,625 miles, making rather a good record, and in all probability it will be required for some time to come.

### OUR LAST TRIP.

We have just returned from a trip to Wonderfontein, some distance east of Middleburg. We entrained 55 privates there, and took on nearly as many more at Middleburg, making 103 in all. Some of the convalescents were able to sit up, but are not ready to return to duty yet, among them being seven Boers. This time the patients were left at No. 7 General Hospital, the siding we are waiting on at present. It was a busy scene when we arrived, ambulances, waggons for kits, no end of stretchers and bearers and medical officers from hospital superintending the moving of the privates. Most of them persist in beginning at the wrong end of the train, or start both at once, much to the distraction of the orderlies. The stretchers are usually placed on a light two-wheel bicycle affair and wheeled up to hospital, making it very easy both for patient and orderly. To-night we take in patients from the three general hospitals, Nos. 2, 7 and 22, bound for Durban. Then after another busy scene for an hour, our visitors will all settle down for the night after being presented with a box of cigarettes. When we reach Durban the patients will be sorted out, those really convalescent and in good condition going home on a transport, the weakly ones and those with chronic complaints sailing on the hospital ship, and so it continues month after month. Enteric fever is very bad on the eastern line at this season in spite of all that is done by the sanitary officers to prevent it, and later on statistics will show if there is any improvement over last year.

### Everybody's Opinion.

[Correspondence on all subjects is invited, but we cannot in any way be responsible for the opinions expressed by our correspondents. No communication can be entertained if the name and address of the correspondent are not given as a guarantee of good faith, but not necessarily for publication. All correspondents should write on one side of the paper only.]

### A NURSE'S EXPERIENCE OF AN EAST END HOME.

"Surgeon" writes: About two months ago a nurse entered an East End nursing home with the object of attending private cases. One day the principal wired to her (she was staying with friends, after having nursed a somewhat anxious case for one month), and sent her to nurse small-pox cases at an isolation hospital. She obeyed this order under protest, as she had not contracted to do this kind of work when she entered the home. After staying a week at the hospital the nurse was feeling ill, and not strong enough to endure the exacting nature of her duties. She therefore requested the sister of the home to allow her to leave, and, on permission being refused, the nurse sent in her resignation, and left the hospital. On applying at the home for her luggage, she met with a curt refusal, and would have found herself stranded in London, had it not been for the kindness of friends. Her property was only restored upon formal application by a solicitor. Now, though possibly the proprietors of this nursing home may have been acting within their rights in sending this young girl to risk her health for their own profit, yet it seems to me they might have shown enough interest in her to take care that she was revaccinated before risking infection; but this they did not do. Nor do I think they acted with even common humanity and justice —to say nothing of that womanly sympathy one would have expected to find in ladies devoted to nursing the sick—in refusing to give up her property. I may add that since leaving the hospital she has been seriously ill. I hope you will find space for this letter, as a warning to nurses that they should certainly insist on special terms with reference to nursing small-pox, and on entering these homes stipulate for a higher rate of pay, on account of the extra risk and the unpleasant nature of such duties.

### OVERWORKED NURSES.

"Matty" alluding to the cheery description and sensible advice given under the above heading on January 25th, writes: Bravo, Nurse Alice! that is the kind of letter which does a nurse good, and it truly shows work done with a cheerful spirit. "Do the next thing" should be the motto for nurses. We get tired, but if one thinks of what one is in a hospital for, how cheerfully one ought to do the work! We do not want in our midst those fine ladies who, because an accident case is brought in and she has half an hour's extra duty (which is made up to her afterwards) cannot go out to dinner or "keep her appointment," etc. We want the true workers, willing to sacrifice much to help. A lady I was nursing showed me a letter from a friend whom she had been instrumental in getting into an hospital, in which she said: "I had to wash a man perfectly well and strong, and Mamma says if I stay I shall lose her respect." The man had dislocated his thigh. Fortunately she left the hospital, and the authorities must have been glad for her to go. As regards time off duty, nurses are far better situated than most working women. Their meal hours and time off duty are regular, and that is why so many delicate women can get through their training. Of course they have their busy days and their slack days. If would-be nurses would only think how well off they are, instead of what short time they have off duty and what long hours of work, how much better and easier they would find things. In private nursing it is different; but one has to meet the requirements of the case, and take into consideration the circumstances. I have been a private nurse since 1894, and have made many friends in all conditions of life. Nurses must be true soldiers, going to the fight cheerfully and patiently, making the best of that which comes in their way.

### A WARNING TO PRIVATE NURSES.

"A London Nurse" writes: I should like to give a few words of warning to private nurses about the danger of using methylated spirit lamps in patients' rooms, and a practical illustration of serious consequences resulting from this may be the best means of impressing this danger upon their minds. I was nursing two small children who were suffering from scarlet fever: one servant had also developed the disease, and the other—being nervous and fearing to get it herself—had decamped, leaving her mistress without anyone to help her to do the work of the house, cooking, etc. Finally, however, she succeeded in getting an old charwoman in, but even this help was insufficient, and made the mother's task a difficult one. The other nurse and I were naturally anxious to give as little trouble as possible, and arranged to boil milk and water in the patients' room by means of a methylated spirit lamp. This answered very well for a time, but one evening when the children were convalescing, after tucking them up for the night, I proceeded, as usual, to light the lamp by which to boil some water, when, without any warning, there was a terrific explosion, and the middle part of the lamp, which held the wick, flew all ablaze on to the window curtains, jets of burning methylated spirit ran along the floor, and in a few seconds the room was enveloped in flames. I saw that it was hopeless to try to put it out before it reached the cots, and, therefore, carried the children into an adjoining room, at the same time shrieking Fire! in order to get help. Fortunately, there were some cyclists passing at the time, and with their help we succeeded in putting out the fire just as three fire-engines arrived, but not before a good deal of damage had been done and a dressing-gown hanging on one of the cots had been burnt. It was a very alarming scene and one which I hope it will never be my lot to see again, but I have thought many times how disastrous it might have been if the patient had been a man and helpless—a typhoid case for instance. It would have been impossible for me to have moved him myself, and he probably would have been burnt before help could have been obtained, to say nothing of the ill effects which must have been the result of shock. Knowing how often one is tempted to use a spirit lamp in a private house, I feel that it will be a kindness to other nurses to let them know of my unpleasant experience, and I would suggest that when a heating apparatus is required for things connected with the patient, an oil-stove should be used in preference to the spirit lamp and that it should be used on a landing, or better still, in a bath-room, and placed inside a large bath. Then, if an accident should occur, the patient will be out of danger, and will not run the risk of shock, which might retard his recovery. One of the children I am writing about was a nervous, highly-strung child, and did not get over the shock for some time, and frequently awoke at night shrieking out that the room was on fire. I learned afterwards from a fireman that half the number of fires which they are called out for originate from the exploding of methylated spirit lamps, and I trust that the relation of my unpleasant experience may be the means of preventing many nurses from using these explosive lamps without first seeing that their patients are removed from danger.

### Death in Our Ranks.

The death of Miss Harriet Thomas, principal of the Medical and Surgical Nursing Home, High Street, Oxford Road, Manchester, is announced. Miss Thomas suffered acutely from Bright's disease for 11 weeks before her death, and was buried in the Southern Cemetery on the 20th of last month, a large number of mourners, including the staff of nurses, following her remains to the grave. She was trained at Manchester Royal Infirmary and St. Mary's Maternity Hospital in that city.

### For Reading to the Sick.

THE VOICE OF GOD.

God, Who speaks to man on every side,
Sending His voices from the outer world,
Glorious in stars, and winds, and flowers, and waves,
And from the inner world of things unseen,
In hopes, and thoughts, and deep assurances,—
Not seldom ceases outward speech awhile,
That the inner, isled in calm, may clearer sound.

*MacDonald.*

---

There are in this loud stunning tide
Of human care and crime,
With whom the melodies abide,
Of th' everlasting chime;
Who carry music in their heart
Through dusky lane and wrangling mart,
Plying their daily task with busier feet,
Because their secret souls a holy strain repeat.

*J. Keble.*

---

Let all creatures be silent in Thy sight, speak Thou alone to me!—*Thos. à Kempis.*

---

Sometimes He speaks in sweet thoughts which come to me, in the tender touches of the Spirit of God in the soul. Sweet and loving Shepherd, all the nobler and better things in me come from Thee. Blessed it is, dear Shepherd, to arouse myself to remember that Thou art speaking to me, for the words that Thou speakest they are spirit and they are life. If He speaks to me, I must listen. How am I to listen for the Divine voice? To listen for Him I must hold the powers of my soul in restraint. I must keep myself in calmness and peace. External things are in movement. Without, is the noise of the world. If this noise is filling my soul, I cannot hear the voice of the Good Shepherd. I *must* keep some time for retirement, for watching over myself, for listening to the voice of the Good Shepherd; then—by His Holy Spirit—He will guide me. If He find me quiet, attentive, listening, then Jesus will teach me. "Speak, Lord, Thy servant heareth."—*Knox-Little.*

---

All these longings and doubts, and this inward distress, are the voice of the Good Shepherd in your heart, seeking to call you out of all that is contrary to His will. Oh, let me entreat of you not to turn away from His gentle pleadings.

*H. W. S.*

---

O Lord, who art our Guide even unto death, grant us, I pray Thee, grace to follow Thee whithersoever Thou goest. In little daily duties to which Thou callest us, bow down our wills to simple obedience, patience under pain or provocation, strict truthfulness of word and manner, humility, kindness; in great acts of duty or perfection, if Thou shouldest call us to them, uplift us to self-sacrifice, heroic courage, laying down of life for Thy truth's sake, or for a brother. Amen.

*C. G. Rossetti.*

---

"Through the thunder comes a human voice
Saying, O heart I made, a heart beats here,
Thou hast no power, nor may'st conceive of Thine;
But love I gave thee, with myself to love
And thou must love Me who has died for thee."

*F. Paget.*

### Notes and Queries.

**The Editor is always willing to answer in this column, without any fee, all reasonable questions, as soon as possible.**

**But the following rules must be carefully observed:—**

**1. Every communication must be accompanied by the name and address of the writer.**

**2. The question must always bear upon nursing, directly or indirectly.**

**If an answer is required by letter a fee of half-a-crown must be enclosed with the note containing the inquiry, and we cannot undertake to forward letters addressed to correspondents making inquiries. It is therefore requested that our readers will not enclose either a stamp or a stamped envelope.**

---

*Bright's Disease.*

(175) Can you tell me if there is a book on Bright's Disease?—*K. S.*

The books on Bright's disease are endless. A nurse will get all she wants if she refers to the chapter dealing with the subject in a book on general medicine, say Taylor or Quain.

*Bathing Male Patients.*

(176) Will you kindly tell me if Poor Law nurses are justified in refusing to bath convalescent male patients?—*A. R.*

We can have no doubt that the bathing of male convalescents, and indeed of all male adults except those who are so seriously ill as to require bathing as part of the treatment, should be done by the porters or other male attendants and not by the female nurses. Whether in an individual case a nurse is justified in refusing to do this work must depend in some degree upon the wording of her agreement with her employers.

*Irosidi.*

(177) I shall feel very much obliged if you will tell me the meaning of the term "Irosidi," and if there is an operation connected with it.—*Sefton Park.*

"Irosidi" is not a term known in medicine.

*D'Arsonval Treatment.*

(178) Will you kindly inform me of the address of the headquarters of the D'Arsonval Treatment in London, and also say if the training is free, or if a premium is required? I should also be glad to know if there are many branches established for administering the treatment.—*C. C. A.*

D'Arsonvalisation is the method of employing "high frequency" currents, as they are called, according to the methods of D'Arsonval. There are neither "headquarters" nor "branches" of such a proceeding. Of course it can only be applied where there is the apparatus. An installation has recently been set up at the Harrogate Baths, but the whole affair is of the nature of an experiment.

*Midwife.*

(179) Will you kindly tell me the best treatment for a case of simple persistent constipation after confinement, when there has been no difficulty beforehand? Is the babe likely to suffer if the nursing mother lives on fruit diet from first?—*A. Papprill, Simla, India.*

If the constipation is so severe as to be regarded as a disease, it is a case for a medical practitioner. It may, however, be here pointed out that the alteration in the mode of life, and the greater confinement in the house, which sometimes occurs after the birth of a baby, may tend to produce constipation, as also may the free use of milk in the diet which some mothers indulge in with the hope of producing good milk, and that in such cases removing the cause may remove the effect. A mixed diet containing oatmeal, green vegetables, wholemeal bread, ripe fruits, stewed figs and plums, and plenty of fatty matter, together with exercise, and, in some cases, massage to the abdomen, will generally overcome any tendency to constipation. But, as we have already said, if the condition is severe, it becomes a medical affair—a local examination may be required. As to the second question, no mother does live exclusively on a fruit diet, but if what is meant is taking fruit with the diet, we should answer that it will do no harm so long as it agrees—*i.e.*, causes neither flatulence, acidity, nor diarrhœa.

**Standard Books of Reference.**

"The Nursing Profession: How and Where to Train." 2s. net; post free 2s. 4d.
"Burdett's Official Nursing Directory." 3s. net; post free, 3s. 4d.
"Burdett's Hospitals and Charities." 5s.
"The Nurses' Dictionary of Medical Terms." 2s.
"Burdett's Series of Nursing Text-Books." 1s. each.
"A Handbook for Nurses." (Illustrated). 5s.
"Nursing: Its Theory and Practice." New Edition. 3s. 6d.
"Helps in Sickness and to Health." Fifteenth Thousand. 5s.
"The Physiological Feeding of Infants." 1s.
"The Physiological Nursery Chart." 1s.; post free, 1s. 3d.
"Hospital Expenditure: The Commissariat." 2s. 6d.
All these are published by the Scientific Press, Ltd., and may be obtained through any bookseller or direct from the publishers 28 and 29 Southampton Street, London, W.C.

### Travel Notes.

By Our Travelling Correspondent.

### XCII.—THROUGH THE CATACOMBS, Etc.

I am sure if my readers have been continental travellers they have many times suffered from the attentions of the "instructive tourist"! Oh! how well I know him; beautiful romantic Fiesole set on its stately hill, is for ever associated in my mind with the terrors of the "instructive" man. I think he is chiefly a horrid product of the 19th century. In bygone years only the wealthy could travel far, and they managed it in a species of aristocratic seclusion, that insured them against the officious attentions of hotel companions, who either are, or at any rate fancy themselves to be better instructed than others, and flood the long suffering if ignorant majority with dry-as-dust items of information that cause the unregenerate to long for a secluded spot in which to turn and rend them. I suffered thus in the catacomb of St. Calixtus. We had made up a small party for the expedition, and at the last moment were kindly informed by the instructive one that "he didn't mind if he joined us"! In some happy positions it is easy to detach oneself from one's companions, but not so in the catacombs, they are so vast, so bewildering, and so mazelike that only absolute safety is insured by the presence of a guide and plenty of "*cerini*" (tapers); it would be highly dangerous to leave the others, however great the temptation. Mr. Baedeker says that if the catacombs were stretched out in single file, as it were, they would extend over 500 miles, and it is easy to imagine it, for the cells for the dead hewn out of the rock and closed with slabs were constructed one above another sometimes in five tiers.

"The intricate passages along which they followed their guide had been hewn, in some forgotten age, out of a dark red crumbly stone. On either side were horizontal niches, where, if they held their torches closely, the shape of a human body was discernible in white ashes, into which the entire mortality of a man or woman had resolved itself. Among all this extinct dust, there might perchance be a thighbone which crumbled at a touch; or, possibly a skull, grinning at its own wretched plight, as is the ugly and empty habit of the thing. . . . Here and there the narrow and tortuous passages widened somewhat, developing themselves into small chapels, which once, no doubt, had been adorned with marble work and lighted with ever-burning lamps and tapers." Hawthorne.

In one of these St. Cecilia's body was found by Pope Paschel in the ninth century. After her ghastly martyrdom in Trastevere, her firm friend Pope Urban caused her to be placed in this catacomb, where she remained 600 years. Paschel transported her body, and those of her husband and brother, to the church of St. Cecilia, and there in the sixteenth century the tomb was again opened, and the beautiful body of the young girl found to be but little changed. It lay on its side as if in sleep, with the knees slightly flexed. Stefano Moderno, the great sculptor of his day, looked upon her with awe and profound admiration, copied what he saw, and gave to the world the lovely figure which now rests on her grave.

I was meditating on all this as I looked on the empty niche in the catacomb, when I was roused by the instructive voice raised loudly so that no visitor should lose any single gem of his erudition. "And so I must remind you that Moderno, somewhere about the year" . . . but armed with my taper I fled to the furthest spot compatible with safety, and so this pearl of information was lost to me for ever.

### The Catacomb of S. Agnese.

To reach this you go out by the Porta Pia along a really frightful road with modern trumpery houses; well may Mr. Hare lament in glowing terms the destruction of the beautiful grounds of the Villa Patrizi with the Judas trees and Ilex. I never saw this loveliness and have only been privileged to behold in its place a dusty, sordid, mean kind of Piazza. Truly nothing is sacred in the way of natural beauty, and very little in that of architectural to the desecrating hand of municipal Rome. About a mile from the city is the church of S. Agnese fuori le Mura, and here, on the occasion of my visit, there was to be an Italian archæological lecture. We went along like Cook's tourists in a band of thirty or forty, first to the small round church of S. Costanza. The roof is adorned with frescoes representing chiefly vintage scenes, and we were told that probably it had originally been a mausoleum and afterwards used as a Baptistery. From there we plodded along more dusty roads to the catacomb, the entrance to which in a field our lecturer had some difficulty in finding. Presently he discovered it, and we descended into the bowels of the earth by steep and ancient steps. We found ourselves in a narrow passage, lighted with feeble candles at intervals, and with shelves all the way up like berths in a steamer, in which were reposing bones, skulls, and dust. Here and there a passage branched off as black as pitch, and leading into seemingly endless darkness. Arrived at a small chapel we sadly listened to a most tedious discourse, the lecturer first giving all the reasons to prove that St. Peter had once preached there, and sat in a very uncomfortable stone chair, and then when we thought it was all satisfactorily settled, turning round and showing that it was impossible. In another small chapel we found some curious paintings—"Daniel in the lion's den," "Jonah and the whale," etc., but between darkness and defacement it was difficult to tell which was Jonah and which the whale. Then, oh joy! it was all over and we could get into the blessed daylight again. As we ascended whom should we encounter, trotting complacently forward, with a devout following of ladies, but the instructive man. He stretched out a detaining hand: "Were we sure we had seen everything? Could he be of any use!" We fled, and only drew breath in the sunny seclusion of our own rooms over a festive cup of tea. There are numerous other catacombs, but I think on the whole those I have mentioned are the best worth seeing, and there is a strong family resemblance between them; when you have seen one you have seen all. One would not willingly leave Rome without seeing at least one of these singular places, but I prefer air and light myself, and above all things I loathe archæological lectures and instructive bores!

### TRAVEL NOTES AND QUERIES.

Vitré for Sketching (Artist).—A delightful place, almost unspoilt by the municipal restorer. The landlady of the Hotel de France will be found charming. It is a small place but full of interest, and if you sketched every day for six months you would not exhaust its resources. The best houses from an artistic point of view are those in the Place de la Halle, but there are also some gems in the back streets near to the water. Vitré bears some resemblance to our own Chester.

Florence for the Winter (Stella).—The nicest route is *viâ* Calais, Laon, Lucerne, and the St. Gothard; this costs, second-class single, £6 4s. 2d. Much cheaper is the route *viâ* Newhaven, Dieppe, Rouen, and Turin—cost, second single, £5 4s. 8d. I should go by the first and return by the second so as to pass by the St. Gothard and the Mont Cenis. When you have decided later on the time of your departure I will send you addresses. Whether you choose winter or later spring will make all the difference in the selection of locality for your pension or hotel. Florence is very cold in the winter but clear and sunny and very dry. Warm light clothing is a necessity. Take everything woollen certainly. Yes, there are charming apartments near the Cascine if you remain long, but for a short stay it is too far from all the sights.

## Figures and Tables

**Fig. 21. f1:**
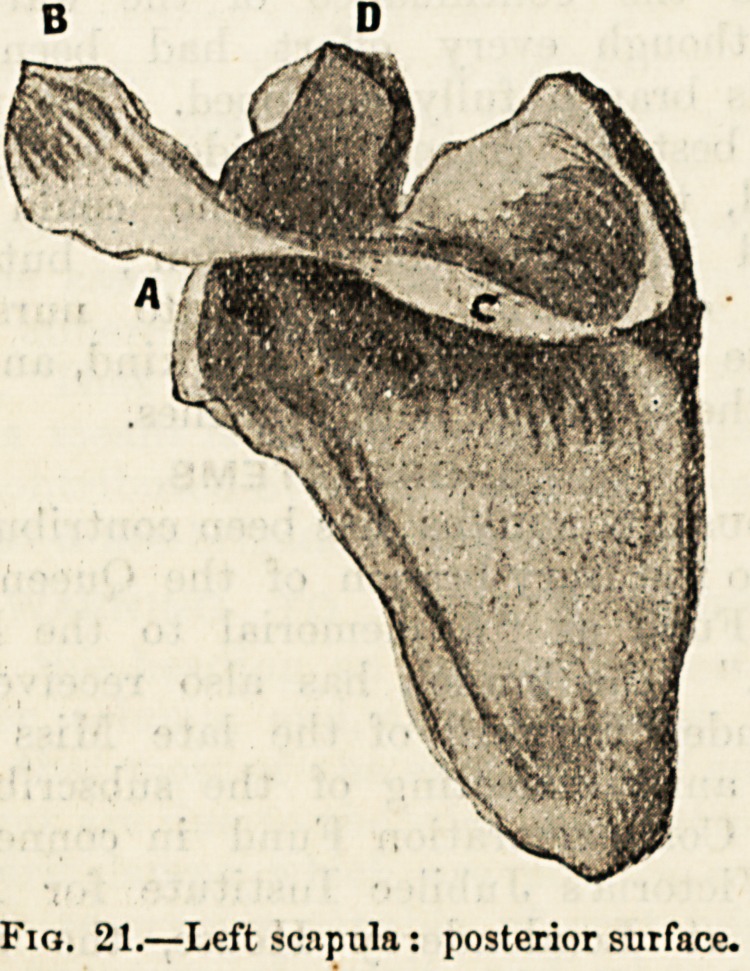


**Fig. 22. f2:**
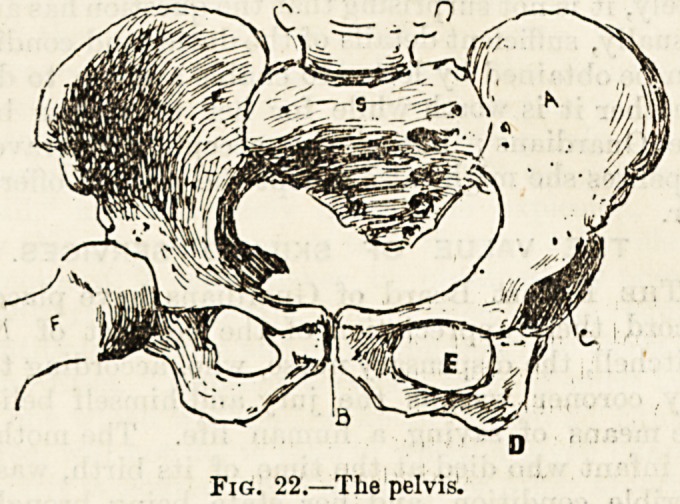


**Fig. 23. f3:**